# Multiplicity-weighted Euler’s formula for symmetrically arranged space-filling polyhedra

**DOI:** 10.1107/S2053273320007093

**Published:** 2020-07-09

**Authors:** Zbigniew Dauter, Mariusz Jaskolski

**Affiliations:** a Macromolecular Crystallography Laboratory, National Cancer Institute, Argonne, IL 60439, USA; b Department of Crystallography, Faculty of Chemistry, A. Mickiewicz University and Center for Biocrystallographic Research, Institute of Bioorganic Chemistry, Polish Academy of Sciences, Poznan, Poland

**Keywords:** asymmetric unit, unit cell, Euler’s formula, space-filling polyhedra, Dirichlet domains

## Abstract

The famous Euler’s rule for three-dimensional polyhedra, *F* − *E* + *V* = 2 (*F, E* and *V* are the numbers of faces, edges and vertices, respectively), when extended to many tested cases of space-filling polyhedra such as the asymmetric unit (ASU), takes the form *Fn* − *En* + *Vn* = 1, where *Fn, En* and *Vn* enumerate the corresponding elements, normalized by their multiplicity, *i.e.* by the number of times they are repeated by the space-group symmetry. This modified formula holds for the ASUs of all 230 space groups and 17 two-dimensional planar groups as specified in the *International Tables for Crystallography*, and for a number of tested Dirichlet domains, suggesting that it may have a general character. The modification of the formula stems from the fact that in a symmetrical space-filling arrangement the polyhedra (such as the ASU) have incomplete bounding elements (faces, edges, vertices), since they are shared (in various degrees) with the space-filling neighbors.

## Abstract 2

For many tested cases of identical space-filling polyhedra, such as the space-group-specific asymmetric units or Dirichlet domains, the numbers of their faces (*Fn*), edges (*En*) and vertices (*Vn*), in each case normalized by division by the multiplicity of their (potentially special) symmetry position, fulfill a modified Euler’s formula *Fn* − *En* + *Vn* = 1.

## 1. Introduction   

The famous Euler’s formula (Euler, 1758 ▸) states that for any three-dimensional polyhedron the sum of the numbers of its faces (*F*) and vertices (*V*) is by two larger than the number of its edges (*E*):
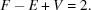
This theorem is the origin of the whole field of topology (*e.g.* Weeks, 1985 ▸; Thurston, 1998 ▸; Nikulin & Shafarevich, 2002 ▸). Euler’s formula can be derived and proven in many ways, *e.g.* as given at https://www.ics.uci.edu/~eppstein/junkyard/euler/.

In the course of an analysis of the relations between the geometrical elements of the asymmetric unit (ASU) of the crystallographic unit cells in different space groups [analogous to the work of Grosse-Kunstleve *et al.* (2011 ▸), http://cci.lbl.gov/asu_gallery/], we realized that another, modified, formula holds for the bounding elements of the ASU.

As defined in the IUCr Online Dictionary of Crystallography (http://reference.iucr.org/dictionary/), an ASU of a space group is ‘*a simply connected smallest closed part of space from which, by application of all symmetry operations of the space group, the whole space is filled*’. The exact selection of the ASU for a particular space group is somewhat arbitrary, and the most convenient choice is an ASU that is a contiguous and convex polyhedron. Such conveniently selected ASUs are defined and presented in the *International Tables for Crystallography*, Vol. A (Aroyo, 2016 ▸) (ITA) for each of the 230 space groups and 17 plane groups. They are addressed in the following section.

## 2. Asymmetric units of the crystallographic space groups   

**Figure 1:**
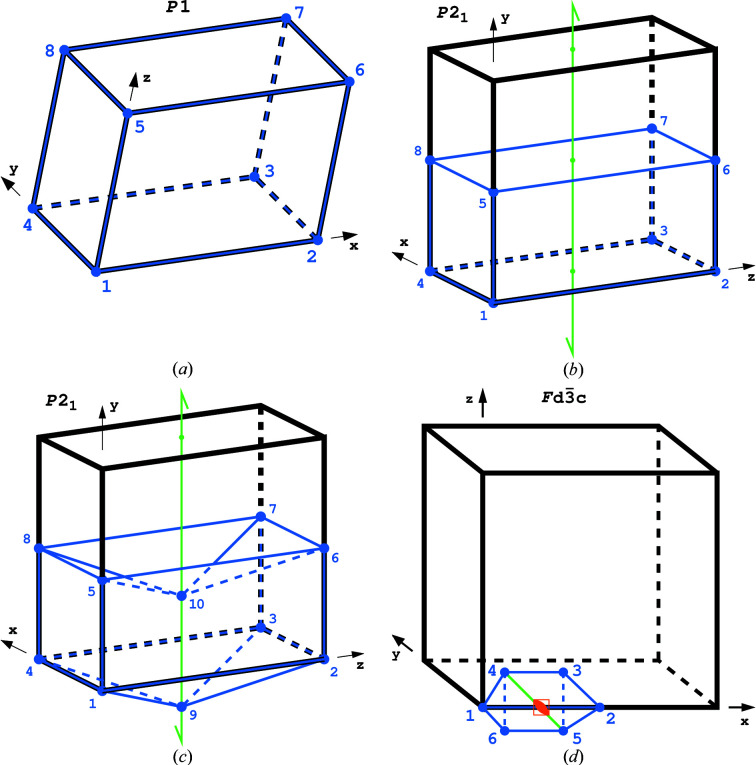
(*a*) The ASU in the space group *P*1, where vertices 2, 3…8 are equivalent by translation to vertex 1, all four edges parallel to a given axis have only one unique member (*e.g.* 4–3, 5–6 and 8–7 are equivalent to 1–2) and only one face from each parallel pair is unique. Black is used to outline the crystallographic unit cell and blue is used to mark the ASU, whose elements coincide with the whole unit cell in this case. (*b*) The ASU (blue) in the unit cell (black) of space group *P*2_1_, where a twofold screw axis (only one is shown in green) transforms the bounding elements of the ASU at *y* = ½ onto the corresponding elements at *y* = 0. See text for detailed explanation. (*c*) The ASU (blue) in the unit cell (black) of space group *P*2_1_, modified by addition of two vertices positioned at the central 2_1_ axis. The symmetry relations between different ASU bounding elements of this ASU, which in this case is a concave polyhedron, are explained in the text. (*d*) The ASU (blue) in the unit cell (black) of space group *Fd* 3 *c*, where a twofold axis transforms one-half of the face 4–3–5–6 onto the other half and the 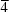 operation centered at *x* = ¼ transforms the edge 1–2 four times onto itself. Moreover, the vertices 1 and 2 lie at the 12-fold redundant special position with 23 symmetry, vertices 3, 4, 5, 6 lie at the sixfold Wyckoff position 32, and the pairs of equivalent edges 1–4/2–5 and 1–6/2–3 lie on the threefold axes. The color code is as in (*a*); in addition the twofold axis is presented in green and the special position at the 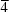 inversion point is marked in orange.

Each ASU is defined in ITA by equations of the limiting planes and sometimes by coordinates of its vertices. However, in all ASUs some pairs of their bounding elements (faces, edges and vertices) are equivalent by the space-group symmetry and, consequently, in rigorous definition only one unique element of each such pair should be included in the strict definition of the ASU. For the simplest example of the space group *P*1 [Fig. 1 ▸(*a*)] the ITA formula 0 ≤ *x* ≤ 1; 0 ≤ *y* ≤ 1; 0 ≤ *z* ≤ 1 defines a complete parallelepiped, whereas in reality all eight corners are equivalent by lattice translations, only one face of each of the three parallel pairs is unique, and the edges in each set of parallel four are equivalent as well. The strict definition of the ASU for this space group should, therefore, be 0 ≤ *x* < 1; 0 ≤ *y* < 1; 0 ≤ *z* < 1, which excludes the redundant elements, leaving only one unique element from each equivalent group. More complicated cases are illustrated in Figs. 1 ▸(*b*), 1 ▸(*c*), 1 ▸(*d*) for the space groups *P*2_1_ and *Fd* 3 *c*, where some of the elements lie at special positions and/or are transformed by symmetry onto themselves or onto other, equivalent and unique elements.

Of course, as all other three-dimensional polyhedra, all crystallographic ASUs must fulfill the Euler’s formula. However, we noticed that they also fulfill a modified rule:
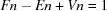
where *Fn*, *En* and *Vn* are, respectively, the numbers of the faces, edges and vertices, in each case divided by their multiplicity or, in other words, by the number of times they are repeated by the space-group symmetry operations.

There are no special positions in the space group *P*1 [Fig. 1 ▸(*a*)]; therefore, for this space group *Fn* = 3 (each of the six faces is repeated twice in pairs), *En* = 3 (three sets of four parallel edges equivalent by translation) and *Vn* = 1 (all eight vertices equivalent). Thus, for *P*1, the modified Euler’s rule 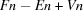 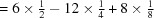 = 1 is obviously fulfilled.

Fig. 1 ▸(*b*) illustrates a possible ASU in the space group *P*2_1_. Here two sets of four vertices at *z* = 0 and *z* = ½ are related by one of the 2_1_ axes, similarly as pairs of parallel horizontal edges and the pair of faces *z* = 0 and *z* = ½. All four vertical edges are equivalent by translation, as are the pairs of parallel vertical faces. In effect, all vertices are equivalent, there are four sets of parallel and equivalent edges and three pairs of equivalent faces. The normalized Euler’s formula is 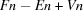 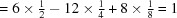.

In Fig. 1 ▸(*c*) the *P*2_1_ case is modified by replacing each of the horizontal faces at *z* = 0 and *z* = ½ with a set of four small pyramidal facets with an apex at the central 2_1_ axis below the level of the original face. Such a concave polyhedron can also serve as the ASU in the space group *P*2_1_. It has an additional pair of equivalent vertices, leading to 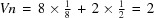; four additional pairs of equivalent edges, leading to 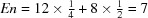; and four additional pairs of equivalent and parallel facets replacing the original equivalent pair, leading to 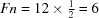. Hence the normalized Euler’s formula also holds for this concave ASU, and is in this case 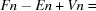 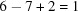.

A high-symmetry example is illustrated in Fig. 1 ▸(*d*) for space group *Fd* 3 *c*. The face 4–3–5–6 transforms into itself by the operation of the twofold axis, and the pairs of faces 1–2–3–4/1–2–5–6 and 1–4–6/2–3–5 are equivalent by the 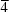 operation, yielding *Fn* = 1 × ½ + 4 × ½ = 2½. Edge 1–2 is positioned along the 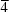 inversion axis and is, therefore, fourfold redundant, four edges (3–4, 3–5, 4–5, 4–6) are equivalent either by a twofold or 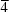 operation, and there are two pairs of edges (1–4/2–5 and 1–6/2–3) oriented along threefold axes that are equivalent by 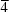. The *En* value is, therefore, 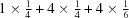 = 23/12. Two equivalent vertices 1 and 2 lie at the 12-fold redundant 23 sites, two equivalent vertices 3 and 6 lie at the sixfold 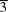 site, and two equivalent vertices 4 and 5 lie at the sixfold 32 site, yielding 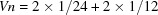 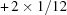 = 5/12. The normalized Euler’s formula 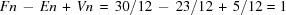 is, therefore, fulfilled as well.

We have analogously interpreted all 230 space groups, and in all cases the modified Euler’s rule holds for their ASUs defined in ITA. The normalized Euler’s parameters *Fn*, *En* and *Vn* for all these space groups are presented in Table S1 in the supporting information.

## 3. Two-dimensional planar groups   

The Euler’s formula for all polygons is 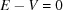, since each polygon always has equal numbers of corners and edges. A similar concept of a normalized Euler’s formula can also be applied to the plane-filling symmetric polygons in the two-dimensional planar groups and can be shown to have the form 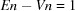.

**Figure 2:**
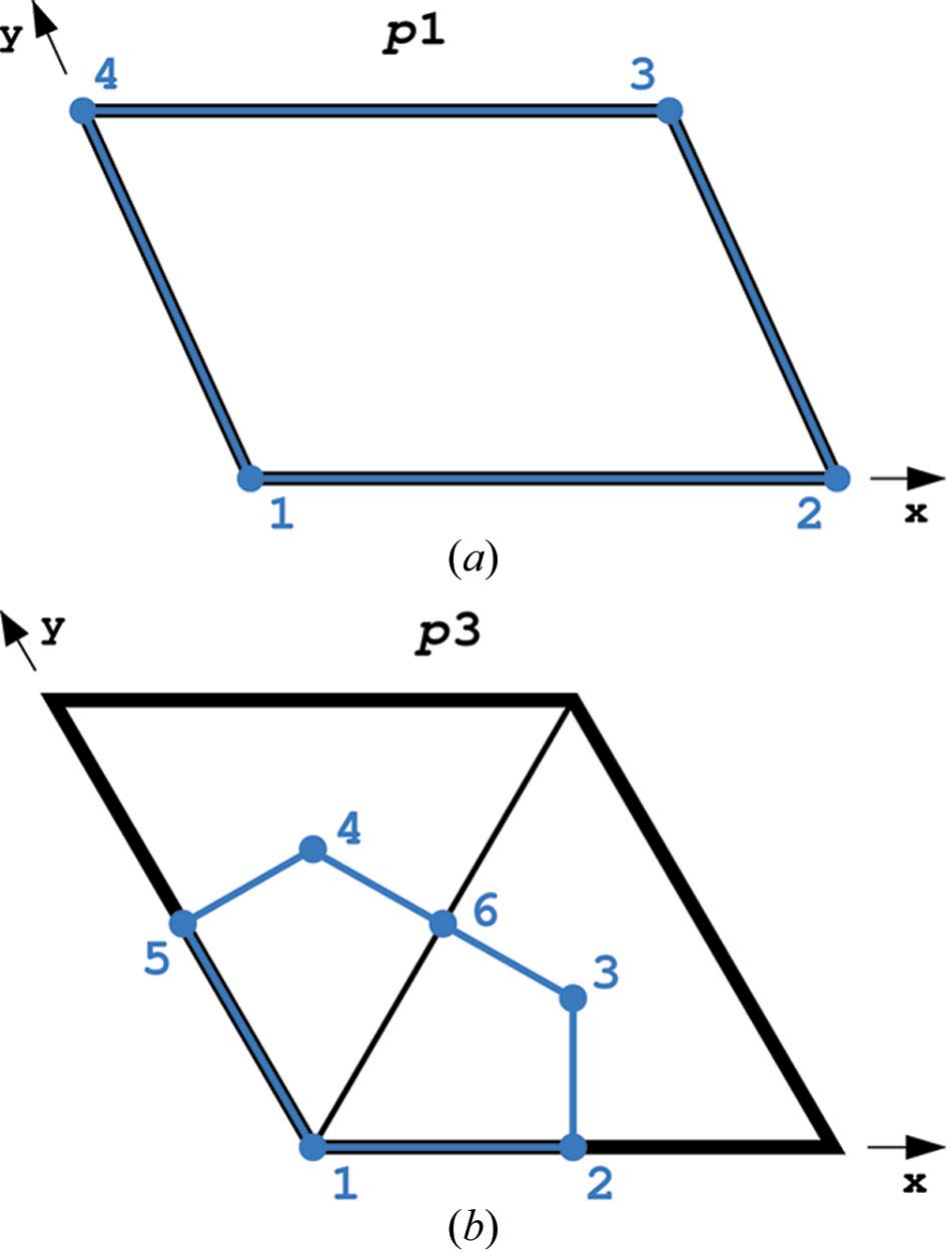
(*a*) The two-dimensional group *p*1 and its ASU (marked in blue) encompassing the whole unit cell (black). All vertices are equivalent by translation and the two edges in each parallel pair are also equivalent. (*b*) The ASU (blue) of the unit cell (black) of the two-dimensional group *p*3. The three vertices 1, 3 and 4 are positioned at the threefold axes. The vertices 2, 5 and 6 are equivalent by the threefold axes. The pairs of edges 1–2/1–5, 3–2/3–6 and 4–5/4–6 are also equivalent by the operation of the threefold axes.

As illustrated in Fig. 2 ▸(*a*), in the two-dimensional group *p*1 each of all four vertices and two edges in each parallel pair delimiting the ASU are equivalent and they are all in general positions. Therefore, 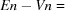 4 × ½ − 4 × ¼ = 1.

In the two-dimensional group *p*3 [Fig. 2 ▸(*b*)] the vertices 1, 3 and 4 lie at the threefold axes, but are not equivalent to each other. Vertices 2, 5 and 6 are equivalent by the threefold rotation axes and lie at general positions. Thus, 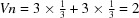 . Edge 1–5 is equivalent to the edge 1–2, and the edges 3–6 and 4–6 are equivalent by the threefold axes to the edges 3–2 and 4–5, respectively. Hence, 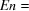 2 × ½ + 4 × ½ = 3. The modified Euler’s formula is, therefore, written as follows: 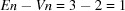. The normalized Euler’s parameters *En* and *Vn* for all 17 planar groups are presented in Table S2.

The normalized Euler’s formula seems to hold also in four dimensions, as illustrated in the appendix in the supporting information for a four-dimensional hyper-parallelepiped.

## 4. The Dirichlet domains   

A specific kind of space-filling polyhedra are the Dirichlet domains, sometimes called the Voronoi polyhedra, regions of influence, or (in mathematics) fundamental regions (Voronoi, 1908 ▸; Delaunay, 1933 ▸; Engel, 1986 ▸). A Dirichlet domain consists of all points that are closer to a selected generating point in a lattice than to any of its space-group-symmetry-equivalent points. Such a domain is thus always a polyhedron bounded by planes normal at half-length to vectors joining the generating point with its neighbors. A Dirichlet domain can, therefore, be treated as a form of the ASU, since it contains only the unique part of the unit cell and the whole space is filled by identical polyhedra without any gaps. In general, Dirichlet domains have more complicated shapes than the ASUs defined in ITA and are less useful in the practice of structural crystallographic computations, but in fact they better correspond with the shapes of (globular) molecules positioned in various places of a crystal unit cell.

By analogy with the previously addressed ASUs, the external elements of Dirichlet domains (faces, edges or vertices) are also often located at the special positions of the unit cell, because the bounding faces lie exactly at the symmetry elements relating the generating point with its symmetry equivalents. It is, therefore, interesting to check if the modified Euler’s formula also holds for the Dirichlet domains. This analysis cannot be fully comprehensive, since the number of all possible topologically different domains in three dimensions is very large and not known, although this number is finite (Delaunay, 1961 ▸).

**Figure 3:**
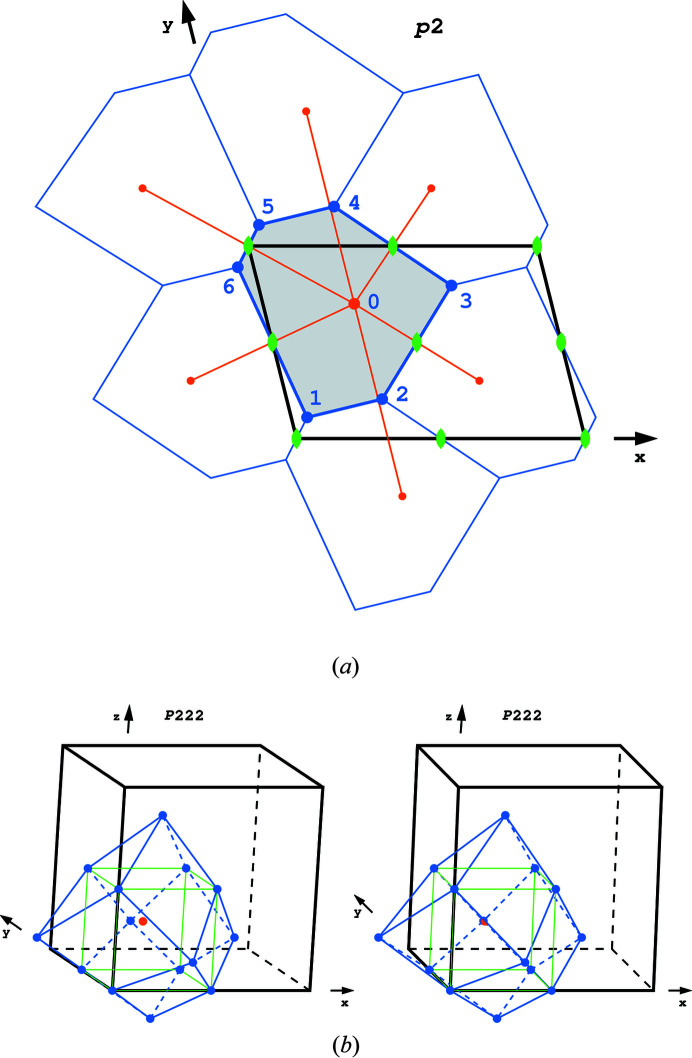
(*a*) The Dirichlet domain around the point *x* = 0.32, *y* = 0.7 in the two-dimensional group *p*2 with cell parameters *a* = 1.45, *b* = 1 (arbitrary units), γ = 76°. The neighboring domains are also shown to emphasize that the plane is filled completely. The generating point and its neighboring symmetry-equivalent points together with the lines joining them are in orange, the meaning of other colors is as in previous figures. (*b*) Stereo presentation of the Dirichlet domain constructed around the point *x* = *y* = 0.2, *z* = 0.3 in the space group *P*222. It has the shape of a distorted rhombic dodecahedron with the same topology, *i.e.* the same number of faces, edges and vertices joined in the same way, as the cubic polyhedron with the same morphology. The twofold axes are shown in green and the generating point is orange.

Fig. 3 ▸(*a*) illustrates a Dirichlet domain for the planar group *p*2 and shows how the domains are formed by planes (in this case by lines) perpendicular to the vectors joining the generating point with its neighbors. The four edges of the domain that lie across the twofold axes are unique, while the two parallel edges of the remaining pair (1–2 and 5–4) are equivalent by translation, yielding *En* = 4 × ½ + 2 × ½ = 3. Among the vertices, there are two triplets (1, 5, 6 and 2, 3, 4) of equivalent ones, related either by translation or by the twofold axes, and *Vn* = 6 × 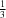 = 2. The normalized Euler’s formula is, therefore, 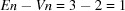.

Fig. 3 ▸(*b*) shows a Dirichlet domain constructed around a point with coordinates 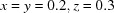 in space group *P*222 with all unit-cell lengths equal. It has the shape of a distorted rhombic dodecahedron and the corresponding topology. All 12 rhombic faces are unique, but they lie at the twofold axes, and thus *Fn* = 12 × ½ = 6. Among the 14 vertices, eight sit at the special 222 positions and are unique, and all six remaining apical vertices are equivalent by some of the twofold axes, so that 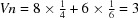 . The three edges crossing at the 222 symmetry position are equivalent by one of the twofold axes, and there are eight such triplets, so that 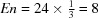. The normalized Euler’s formula is, therefore, 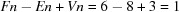.

We checked several other Dirichlet domains in different space and planar groups and they all agree with the normalized Euler’s formula, behaving in the same way as the above-analyzed ASUs of all space and planar groups from the ITA.

## 5. Conclusions   

The applicability of the modified (normalized) Euler’s formula to space-filling polyhedra with symmetrical restrictions on their bounding elements (faces, edges, vertices) is somewhat puzzling to us, structural crystallographers, who constantly utilize the concept of the asymmetric unit in our practice of structural chemistry and biology. We are curious if more qualified topologists will be able to provide a rigorous mathematical proof of the general correctness of this rule, so far confirmed to hold by exhaustive enumeration for all examples of crystallographic two-dimensional and three-dimensional groups.

## Supplementary Material

Tables of normalized Euler's parameters for space and planar groups, and appendix. DOI: 10.1107/S2053273320007093/sc5138sup1.pdf
