# Supplementary material for: Multiplicity-weighted Euler’s formula for symmetrically arranged space-filling polyhedra
Source: Acta Crystallogr A Found Adv. 2020 Jul 9;76(Pt 5):580–3. doi: 10.1107/S2053273320007093 (PMC7459769; doi:10.1107/S2053273320007093)
Supplement: Supplementary file 1 [file a-76-00580-sup1.pdf]

FOUNDATIONS
ADVANCES

Volume 76 (2020)

Supporting information for article:

Multiplicity-weighted Euler's formula for symmetrically arranged space-filling polyhedra

Zbigniew Dauter and Mariusz Jaskolski

Table S1. Original and normalized Euler's formulae for the asymmetric units specified for all three-dimensional space groups in the *International Tables for Crystallography*, vol. A (2016). The symbol ":*i*" (*i*=1 or 2) designates different choice of origin.

Space group		Euler's formula				Normalized formula			
No	Symbol	F +	V -	E =	D	Fn +	Vn -	En =	Dn
1	$P1$	6	8	12	2	3	1	3	1
2	$P\bar{1}$	8	8	14	2	4	2	5	1
3	$P2$	8	12	18	2	3	1	3	1
4	$P2_1$	8	12	18	2	4	2	5	1
5	$C2$	8	12	18	2	4	2	5	1
6	Pm	6	8	12	2	5	4	8	1
7	Pc	8	12	18	2	4	2	5	1
8	Cm	7	10	15	2	$3\frac{1}{2}$	$1\frac{1}{2}$	4	1
9	Cc	10	15	23	2	5	3	7	1
10	$P2/m$	8	12	18	2	4	2	5	1
11	$P2_1/m$	6	8	12	2	$3\frac{1}{2}$	$1\frac{1}{2}$	4	1
12	$C2/m$	8	12	18	2	$4\frac{1}{2}$	$2\frac{1}{2}$	6	1
13	$P2/c$	8	12	18	2	4	2	5	1
14	$P2_1/c$	8	10	16	2	4	2	5	1
15	$C2/c$	10	12	20	2	5	3	7	1
16	$P222$	10	12	20	2	5	2	6	1
17	$P222_1$	14	20	32	2	7	4	10	1
18	$P2_12_12$	6	8	12	2	3	1	3	1

19	$P2_12_12_1$	10	12	20	2	4	2	5	1
20	$C222_1$	8	12	18	2	4	2	5	1
21	$C222$	10	14	22	2	5	$2\frac{1}{2}$	$6\frac{1}{2}$	1
22	$F222$	14	18	30	2	7	$3\frac{1}{2}$	$9\frac{1}{2}$	1
23	$I222$	6	8	12	2	3	1	3	1
24	$I2_12_12_1$	12	20	30	2	6	4	9	1
25	$Pmm2$	6	8	12	2	3	1	3	1
26	$Pmc2_1$	8	12	18	2	4	2	5	1
27	$Pcc2$	10	12	20	2	5	2	6	1
28	$Pma2$	7	10	15	2	$3\frac{1}{2}$	$1\frac{1}{2}$	4	1
29	$Pca2_1$	8	12	18	2	4	2	5	1
30	$Pnc2$	10	12	20	2	5	2	6	1
31	$Pmn2_1$	8	12	18	2	4	2	5	1
32	$Pba2$	10	16	24	2	5	3	7	1
33	$Pna2_1$	8	12	18	2	4	2	5	1
34	$Pnn2$	10	12	20	2	5	2	6	1
35	$Cmm2$	7	10	15	2	$3\frac{1}{2}$	$1\frac{1}{2}$	4	1
36	$Cmc2_1$	6	8	12	2	3	1	3	1
37	$Ccc2$	10	14	22	2	5	$2\frac{1}{2}$	$6\frac{1}{2}$	1
38	$Amm2$	6	8	12	2	3	1	3	1
39	$Abm2$	9	12	19	2	$4\frac{1}{2}$	2	$5\frac{1}{2}$	1
40	$Ama2$	9	12	19	2	$4\frac{1}{2}$	2	$5\frac{1}{2}$	1
41	$Aba2$	6	8	12	2	3	1	3	1
42	$Fmm2$	8	11	17	2	4	$1\frac{3}{4}$	$4\frac{3}{4}$	1
43	$Fdd2$	18	20	36	2	9	4	12	1

44	*Imm*2	6	8	12	2	3	1	3	1
45	*Iba*2	6	8	12	2	3	1	3	1
46	*Ima*2	9	12	19	2	$4\frac{1}{2}$	2	$5\frac{1}{2}$	1
47	*Pmmm*	6	8	12	2	3	1	3	1
48	*Pnnn*:1	12	16	26	2	6	3	8	1
48	*Pnnn*:2	16	22	36	2	8	$4\frac{1}{2}$	$11\frac{1}{2}$	1
49	*Pccm*	10	12	20	2	5	2	6	1
50	*Pban*:1	8	8	14	2	5	2	6	1
50	*Pban*:2	8	8	14	2	8	4	11	1
51	*Pmma*	7	10	15	2	$3\frac{1}{2}$	$1\frac{1}{2}$	4	1
52	*Pnna*	12	18	28	2	6	$3\frac{1}{2}$	$8\frac{1}{2}$	1
53	*Pmna*	8	12	18	2	4	2	5	1
54	*Pcca*	12	16	26	2	6	3	8	1
55	*Pbam*	6	8	12	2	3	1	3	1
56	*Pbbn*	12	16	26	2	6	3	8	1
57	*Pbcm*	11	16	25	2	$5\frac{1}{2}$	3	$7\frac{1}{2}$	1
58	*Pnnm*	6	8	12	2	3	1	3	1
59	*Pmmn*:1	8	8	14	2	4	2	5	1
59	*Pmmn*:2	8	8	14	2	$3\frac{1}{2}$	$1\frac{1}{2}$	4	1
60	*Pbcn*	8	12	18	2	4	2	5	1
61	*Pbca*	6	8	12	2	3	1	3	1
62	*Pnma*	9	12	19	2	$4\frac{1}{2}$	2	$5\frac{1}{2}$	1
63	*Cmcm*	7	8	13	2	$3\frac{1}{2}$	$12\frac{1}{2}$	4	1
64	*Cmca*	7	10	15	2	$4\frac{1}{2}$	$2\frac{1}{2}$	6	1
65	*Cmmm*	7	10	15	2	$3\frac{1}{2}$	$2\frac{1}{2}$	5	1

66	*Cccm*	10	14	22	2	5	$2\frac{1}{2}$	$6\frac{1}{2}$	1
67	*Cmma*	9	12	19	2	$4\frac{1}{2}$	2	$5\frac{1}{2}$	1
68	*Ccca*:1	8	10	16	2	4	2	5	1
68	*Ccca*:2	8	10	16	2	5	$2\frac{1}{2}$	$6\frac{1}{2}$	1
69	*Fmmm*	8	11	17	2	4	$1\frac{3}{4}$	$4\frac{3}{4}$	1
70	*Fddd*:1	14	20	32	2	7	$4\frac{1}{2}$	$10\frac{1}{2}$	1
70	*F*ddd:2	16	26	40	2	8	$5\frac{1}{2}$	$12\frac{1}{2}$	1
71	*Immm*	7	8	13	2	$3\frac{1}{2}$	$1\frac{1}{2}$	4	1
72	*Ibam*	10	12	20	2	5	$2\frac{3}{4}$	$6\frac{3}{4}$	1
73	*Ibca*	10	14	22	2	5	$2\frac{1}{2}$	$6\frac{1}{2}$	1
74	*Imma*	10	15	23	2	5	$2\frac{3}{4}$	$6\frac{3}{4}$	1
75	$P4$	6	8	12	2	3	1	3	1
76	$P4_1$	18	20	36	2	9	4	12	1
77	$P4_2$	10	12	20	2	5	2	6	1
78	$P4_3$	18	20	36	2	9	4	12	1
79	$I4$	6	8	12	2	3	1	3	1
80	$P4_1$	10	12	20	2	5	2	6	1
81	$P\bar{4}$	6	8	12	2	3	1	3	1
82	$I\bar{4}$	6	8	12	2	3	1	3	1
83	$P4/m$	6	8	12	2	3	1	3	1
84	$P4_2/m$	6	8	12	2	3	1	3	1
85	$P4/n$:1	8	8	14	2	3	1	3	1
85	$P4/n$:2	8	8	14	2	3	1	3	1
86	$P4_2/n$:1	12	14	24	2	6	3	8	1
86	$P4_2/n$:2	12	14	24	2	4	1	4	1

87	*I*4/*m*	7	8	13	2	$3\frac{1}{2}$	1	$3\frac{1}{2}$	1
88	$I4_1/a$:1	12	17	27	2	6	$1\frac{1}{4}$	$8\frac{1}{4}$	1
88	$I4_1/a$:2	14	18	30	2	7	$3\frac{1}{2}$	$9\frac{1}{2}$	1
89	*P*422	8	8	14	2	4	1	4	1
90	$P42_12$	8	8	14	2	4	1	4	1
91	$P4_122$	8	10	16	2	4	$1\frac{1}{2}$	$4\frac{1}{2}$	1
92	$P4_12_12$	10	13	21	2	5	$2\frac{1}{2}$	$6\frac{1}{2}$	1
93	$P4_222$	12	12	22	2	6	2	7	1
94	$P4_22_12$	8	8	14	2	4	1	4	1
95	$P4_322$	8	10	16	2	4	$1\frac{1}{2}$	$4\frac{1}{2}$	1
96	$P4_32_12$	13	10	21	2	5	$2\frac{1}{2}$	$6\frac{1}{2}$	1
97	*I*422	8	8	14	2	4	1	4	1
98	$I4_122$	14	16	28	2	7	3	9	1
99	*P*4*mm*	5	6	9	2	$2\frac{1}{2}$	$\frac{1}{2}$	2	1
100	*P*4*bm*	5	6	9	2	$2\frac{1}{2}$	$\frac{1}{2}$	2	1
101	$P4_2cm$	7	9	14	2	$3\frac{1}{2}$	1	$3\frac{1}{2}$	1
102	$P4_2nm$	7	9	14	2	$3\frac{1}{2}$	1	$3\frac{1}{2}$	1
103	*P*4*cc*	6	8	12	2	3	1	3	1
104	*P*4*nc*	6	8	12	2	3	1	3	1
105	$P4_2mc$	6	8	12	2	3	1	3	1
106	$P4_2bc$	6	8	12	2	3	1	3	1
107	*I*4*mm*	5	6	9	2	$2\frac{1}{2}$	$\frac{1}{2}$	2	1
108	*I*4*cm*	5	6	9	2	$2\frac{1}{2}$	$\frac{1}{2}$	2	1
109	$I4_1md$	6	8	12	2	3	1	3	1

110	$I4_1cd$	6	8	12	2	3	1	3	1
111	$P\bar{4}2m$	7	9	14	2	$3\frac{1}{2}$	1	$3\frac{1}{2}$	1
112	$P\bar{4}2c$	10	12	20	2	5	2	6	1
113	$P\bar{4}2_1m$	5	6	9	2	$2\frac{1}{2}$	$\frac{1}{2}$	2	1
114	$P\bar{4}2_1c$	6	8	12	2	3	1	3	1
115	$P\bar{4}m2$	8	8	14	2	4	1	4	1
116	$P\bar{4}c2$	12	12	22	2	6	2	7	1
117	$P\bar{4}b2$	8	8	14	2	4	1	4	1
118	$P\bar{4}n2$	12	12	22	2	6	2	7	1
119	$I\bar{4}m2$	8	8	14	2	4	1	4	1
120	$I\bar{4}c2$	8	8	14	2	4	1	4	1
121	$I\bar{4}2m$	5	6	9	2	$2\frac{1}{2}$	$\frac{1}{2}$	2	1
122	$I\bar{4}2d$	12	16	26	2	6	3	8	1
123	$P4/mmm$	5	6	9	2	$2\frac{1}{2}$	$\frac{1}{2}$	2	1
124	$P4/mcc$	7	8	13	2	$3\frac{1}{2}$	1	$3\frac{1}{2}$	1
125	$P4/nbm$:1	7	8	13	2	$3\frac{1}{2}$	1	$3\frac{1}{2}$	1
125	$P4/nbm$:2	7	8	13	2	$3\frac{1}{2}$	1	$3\frac{1}{2}$	1
126	$P4/nnc$:1	7	8	13	2	$3\frac{1}{2}$	1	$3\frac{1}{2}$	1
126	$P4/nnc$:2	7	8	13	2	$3\frac{1}{2}$	1	$3\frac{1}{2}$	1
127	$P4/mbm$	5	6	9	2	$2\frac{1}{2}$	½	2	1
128	$P4/mnc$	7	8	13	2	$3\frac{1}{2}$	1	$3\frac{1}{2}$	1
129	$P4/nmm$:1	7	8	13	2	$3\frac{1}{2}$	1	$3\frac{1}{2}$	1
129	$P4/nmm$:2	7	8	13	2	$3\frac{1}{2}$	1	$3\frac{1}{2}$	1
130	$P4/ncc$:1	7	8	13	2	$3\frac{1}{2}$	1	$3\frac{1}{2}$	1

130	*P*4/*ncc*:2	7	8	13	2	$3\frac{1}{2}$	1	$3\frac{1}{2}$	1
131	*P*4$_2$/*mm*c	7	8	13	2	$3\frac{1}{2}$	1	$3\frac{1}{2}$	1
132	*P*4$_2$/*mcm*	7	9	14	2	$3\frac{1}{2}$	1	$3\frac{1}{2}$	1
133	*P*4$_2$/*nbc*:1	7	8	13	2	$3\frac{1}{2}$	1	$3\frac{1}{2}$	1
133	*P*4$_2$/*nbc*:2	7	8	13	2	$3\frac{1}{2}$	1	$3\frac{1}{2}$	1
134	*P*4$_2$/*nnm*:1	10	10	18	2	5	$1\frac{1}{2}$	$5\frac{1}{2}$	1
134	*P*4$_2$/*nnm*:2	10	10	18	2	$4\frac{1}{2}$	$1\frac{1}{2}$	5	1
135	*P*4$_2$/*mbc*	7	8	13	2	$3\frac{1}{2}$	1	$3\frac{1}{2}$	1
136	*P*4$_2$/*mnm*	7	9	14	2	$3\frac{1}{2}$	1	$3\frac{1}{2}$	1
137	*P*4$_2$/*nmc*:1	7	8	13	2	$3\frac{1}{2}$	1	$3\frac{1}{2}$	1
137	*P*4$_2$/*nmc*:2	7	8	13	2	$3\frac{1}{2}$	1	$3\frac{1}{2}$	1
138	*P*4$_2$/*ncm*:1	9	13	20	2	$4\frac{1}{2}$	$1\frac{3}{4}$	$5\frac{1}{4}$	1
138	*P*4$_2$/*ncm*:2	9	13	20	2	$3\frac{1}{2}$	1	$3\frac{1}{2}$	1
139	*I*4/*mmm*	6	7	11	2	3	$\frac{3}{4}$	$2\frac{3}{4}$	1
140	*I*4/*mcm*	7	8	13	2	3	$\frac{3}{4}$	$2\frac{3}{4}$	1
141	*I*4$_1$/*amd*:1	8	10	16	2	4	$1\frac{1}{2}$	$4\frac{1}{2}$	1
141	*I*4$_1$/*amd*:2	8	10	16	2	4	$1\frac{1}{2}$	$4\frac{1}{2}$	1
142	*I*4$_1$/*acd*:1	8	10	16	2	4	$1\frac{1}{2}$	$4\frac{1}{2}$	1
142	*I*4$_1$/*acd*:2	8	10	16	2	4	$1\frac{1}{2}$	$4\frac{1}{2}$	1
143	*P*3	8	12	18	2	4	2	5	1
144	*P*3$_1$	8	8	14	2	4	1	4	1
145	*P*3$_2$	8	8	14	2	4	1	4	1
146	*H*3	30	22	50	2	15	6	20	1

146	$R3$	6	5	9	2	3	$\frac{1}{3}$	$2\frac{1}{3}$	1
147	$P\bar{3}$	10	12	20	2	5	2	6	1
148	$H\bar{3}$	30	22	50	2	15	6	20	1
148	$R\bar{3}$	6	5	9	2	3	$\frac{1}{3}$	$2\frac{1}{3}$	1
149	$P312$	14	12	24	2	7	2	8	1
150	$P321$	10	12	20	2	5	2	6	1
151	$P3_112$	12	12	22	2	6	2	7	1
152	$P3_121$	8	8	14	2	4	1	4	1
153	$P3_212$	10	10	18	2	5	$1\frac{1}{2}$	$5\frac{1}{2}$	1
154	$P3_221$	8	8	14	2	4	1	4	1
155	$H32$	30	22	50	2	15	6	20	1
155	$R32$	8	9	15	2	4	$\frac{5}{6}$	$3\frac{5}{6}$	1
156	$P3m1$	5	6	9	2	$2\frac{1}{2}$	$\frac{1}{2}$	2	1
157	$P31m$	6	8	12	2	3	1	3	1
158	$P3c1$	14	12	24	2	7	2	8	1
159	$P31c$	10	12	20	2	5	2	6	1
160	$H3m$	5	6	9	2	$2\frac{1}{2}$	$\frac{1}{2}$	2	1
160	$R3m$	4	4	6	2	2		$1\frac{1}{6}$	1
161	$H3c$	30	22	50	2	15	6	20	1
161	$R3c$	10	10	18	2	5	$1\frac{1}{3}$	$5\frac{1}{3}$	1
162	$P\bar{3}1m$	8	8	14	2	4	1	4	1
163	$P\bar{3}1c$	12	12	22	2	6	2	7	1
164	$P\bar{3}m1$	6	8	12	2	3	$\frac{2}{3}$	$2\frac{2}{3}$	1
165	$P\bar{3}c1$	10	12	20	2	5	2	6	1
166	$H\bar{3}m$	7	8	13	2	$3\frac{1}{2}$	1	$3\frac{1}{2}$	1

166	$R\bar{3}m$	6	7	11	2	3	$\frac{2}{3}$	$2\frac{2}{3}$	1
167	$H\bar{3}c$	30	22	50	2	15	6	20	1
167	$R\bar{3}c$	10	8	16	2	5	$\frac{5}{6}$	$4\frac{5}{6}$	1
168	$P6$	6	8	12	2	3	1	3	1
169	$P6_1$	8	8	14	2	4	1	4	1
170	$P6_5$	8	8	14	2	4	1	4	1
171	$P6_2$	8	12	18	2	4	2	5	1
172	$P6_4$	8	12	18	2	4	2	5	1
173	$P6_3$	10	12	20	2	5	2	6	1
174	$P\bar{6}$	8	12	18	2	4	2	5	1
175	$P6/m$	6	8	12	2	3	1	3	1
176	$P6_3/m$	10	12	20	2	5	2	6	1
177	$P622$	8	8	14	2	4	1	4	1
178	$P6_122$	10	10	18	2	5	$1\frac{1}{2}$	$5\frac{1}{2}$	1
179	$P6_522$	8	8	14	2	4	1	4	1
180	$P6_222$	10	12	20	2	5	2	6	1
181	$P6_422$	10	12	20	2	5	2	6	1
182	$P6_322$	12	12	22	2	6	2	7	1
183	$P6mm$	5	6	9	2	$2\frac{1}{2}$	$\frac{1}{2}$	2	1
184	$P6cc$	8	8	14	2	4	1	4	1
185	$P6_3cm$	8	8	14	2	4	1	4	1
186	$P6_3mc$	6	8	12	2	3	$\frac{5}{6}$	$2\frac{5}{6}$	1
187	$P\bar{6}m2$	5	6	9	2	$2\frac{1}{2}$	$\frac{1}{2}$	2	1
188	$P\bar{6}c2$	11	12	21	2	$5\frac{1}{2}$	2	$6\frac{1}{2}$	1
189	$P\bar{6}2m$	6	8	12	2	3	1	3	1

190	$P\bar{6}2c$	9	12	19	2	$4\frac{1}{2}$	2	$5\frac{1}{2}$	1
191	$P6/mmm$	5	6	9	2	$2\frac{1}{2}$	$\frac{1}{2}$	2	1
192	$P6/mcc$	7	8	13	2	$3\frac{1}{2}$	1	$3\frac{1}{2}$	1
193	$P6/mcm$	7	8	13	2	$3\frac{1}{2}$	1	$3\frac{1}{2}$	1
194	$P6/mmc$	6	6	10	2	3	$\frac{3}{4}$	$2\frac{3}{4}$	1
195	$P23$	8	7	13	2	4	$\frac{2}{3}$	$3\frac{2}{3}$	1
196	$F23$	6	5	9	2	3	$\frac{1}{3}$	$2\frac{1}{3}$	1
197	$I23$	6	5	9	2	3	$\frac{1}{3}$	$2\frac{1}{3}$	1
198	$P2_13$	12	10	20	2	6	$1\frac{2}{3}$	$6\frac{2}{3}$	1
199	$I2_13$	10	11	19	2	5	$1\frac{5}{6}$	$5\frac{5}{6}$	1
200	$Pm\bar{3}$	5	5	8	2	$2\frac{1}{2}$	$\frac{1}{3}$	$1\frac{5}{6}$	1
201	$Pn\bar{3}$:1	6	5	9	2	3	$\frac{1}{3}$	$2\frac{1}{3}$	1
201	$Pn\bar{3}$:2	6	5	9	2	3	$\frac{2}{3}$	$2\frac{2}{3}$	1
202	$Fm\bar{3}$	10	15	23	2	$2\frac{1}{2}$	$\frac{5}{12}$	$1\frac{11}{12}$	1
203	$Fd\bar{3}$:1	4	4	6	2	2	$\frac{1}{6}$	$1\frac{1}{6}$	1
203	$Fd\bar{3}$:2	4	4	6	2	2	$\frac{1}{6}$	$1\frac{1}{6}$	1
204	$Im\bar{3}$	4	4	6	2	2	$\frac{1}{6}$	$1\frac{1}{6}$	1
205	$Pa\bar{3}$	6	5	9	2	3	$\frac{1}{3}$	$2\frac{1}{3}$	1
206	$Ia\bar{3}$	6	7	11	2	3	$\frac{5}{6}$	$2\frac{5}{6}$	1
207	$P432$	6	5	19	2	3	$\frac{1}{3}$	$2\frac{1}{3}$	1
208	$P4_232$	12	8	18	2	6	$1\frac{1}{6}$	$6\frac{1}{6}$	1
209	$F432$	10	15	23	2	3	$\frac{5}{12}$	$2\frac{5}{12}$	1
210	$F4_123$	10	8	16	2	5	$\frac{5}{6}$	$4\frac{5}{6}$	1

211	$I432$	8	7	13	2	4	$\frac{7}{12}$	$3\frac{7}{12}$	1
212	$P4_332$	10	15	23	2	5	$1\frac{1}{6}$	$5\frac{1}{6}$	1
213	$P4_132$	10	15	23	2	5	$1\frac{1}{6}$	$5\frac{1}{6}$	1
214	$I4_132$	14	12	24	2	7	$1\frac{5}{6}$	$7\frac{5}{6}$	1
215	$P\bar{4}3m$	5	5	8	2	$2\frac{1}{2}$	$\frac{1}{3}$	$1\frac{5}{6}$	1
216	$F\bar{4}3m$	4	4	6	2	2	$\frac{1}{6}$	$1\frac{1}{6}$	1
217	$I\bar{4}3m$	4	4	6	2	2	$\frac{1}{6}$	$1\frac{1}{6}$	1
218	$P\bar{4}3n$	6	5	9	2	3	$\frac{1}{3}$	$2\frac{1}{3}$	1
219	$F\bar{4}3c$	10	15	23	2	2	$\frac{1}{6}$	$1\frac{1}{6}$	1
220	$I\bar{4}3d$	10	9	17	2	5	$2\frac{1}{3}$	$5\frac{1}{3}$	1
221	$Pm\bar{3}m$	4	4	6	2	2	$\frac{1}{6}$	$1\frac{1}{6}$	1
222	$Pn\bar{3}n$:1	4	4	6	2	2	$\frac{1}{6}$	$1\frac{1}{6}$	1
222	$Pn\bar{3}n$:2	4	4	6	2	2	$\frac{1}{6}$	$1\frac{1}{6}$	1
223	$Pm\bar{3}n$	7	7	12	2	$3\frac{1}{2}$	$\frac{7}{12}$	$3\frac{1}{12}$	1
224	Pn$\bar{3}$m:1	7	6	11	2	$3\frac{1}{2}$	$\frac{7}{12}$	$3\frac{1}{12}$	1
224	$Pn\bar{3}m$:2	7	6	11	2	$3\frac{1}{2}$	$\frac{7}{12}$	$3\frac{1}{12}$	1
225	$Fm\bar{3}m$	4	4	6	2	2	$\frac{5}{24}$	$1\frac{5}{24}$	1
226	$Fm\bar{3}c$	10	15	23	2	$2\frac{1}{2}$	⅓	$1\frac{5}{6}$	1
227	$Fd\bar{3}m$:1	6	6	10	2	3	$\frac{5}{12}$	$2\frac{5}{12}$	1
227	$Fd\bar{3}m$:2	6	6	10	2	3	$\frac{5}{12}$	$2\frac{5}{12}$	1
228	$Fd\bar{3}c$:1	6	6	10	2	3	$\frac{5}{12}$	$2\frac{5}{12}$	1
228	$Fd\bar{3}c$:2	6	6	10	2	3	$\frac{5}{12}$	$2\frac{5}{12}$	1
229	$Im\bar{3}m$	5	5	8	2	$2\frac{1}{2}$	$\frac{7}{24}$	$1\frac{19}{24}$	1

230	$Ia\bar{3}d$	18	17	33	2	9	$3\frac{5}{6}$	$11\frac{5}{6}$	1

Table S2. Original and normalized Euler's formulae for the asymmetric units specified for all two-dimensional space groups in the *International Tables for Crystallography*, vol. A (2016).

Planar group		Euler's formula			Normalized formula		
No	Symbol	E -	V =	D	En -	Vn =	Dn
1	*p*1	4	4	0	2	1	1
2	*p*2	6	6	0	3	2	1
3	*pm*	4	4	0	2	1	1
4	*pg*	6	6	0	3	2	1
5	*cm*	4	4	0	2	1	1
6	*p*2*mm*	4	4	0	2	1	1
7	*p*2*mg*	5	5	0	$2\frac{1}{2}$	$1\frac{1}{2}$	1
8	*pgg*	4	4	0	2	1	1
9	*c*2*mm*	5	5	0	$2\frac{1}{2}$	$1\frac{1}{2}$	1
10	*p*4	4	4	0	2	1	1
11	*p*4*mm*	4	4	0	$2\frac{1}{2}$	$1\frac{1}{2}$	1
12	*p*4*gm*	4	4	0	2	1	1
13	*p*3	6	6	0	3	2	1
14	*p*3*m*1	3	3	0	$1\frac{1}{2}$	$\frac{1}{2}$	1
15	*p*31*m*	4	4	0	2	1	1
16	*p*6	4	4	0	2	1	1

17	*p*6*mm*	3	3	0	$1\frac{1}{2}$	$\frac{1}{2}$	1

Appendix

Normalized Euler's formulae for line segment, parallelogram, parallelepiped and 4-dimensional solid

In **1-dimensional lattice**, the periodically repeated element is the line segment with vertices at coordinates x=0 and x=1. The Euler's formula in 1D is V=2.
These two vertices are equivalent by the lattice translation, therefore their weight in the normalized Euler's formula in 1D is ½, and Vn=2×½=1.

In **2-dimensional plane**, the parallelogram has four vertices at the following points: (x,y)=V1: (0,0), V2: (0,1), V3: (1,0), V4: (1,1). They are all equivalent by translations and their weight in the normalized Euler's formula is ¼.
Out of the six lines connecting pairs these vertices, four constitute the external, bounding edges (not the internal diagonals) that have one coordinate equal for both vertices. Their lengths along the x and y axes are: E1=V1-V2=(0,1), E2=V1-V3=(1,0), E3=V2-V4=(1,0), E4=V3-V4=(0,1).
Edges E1 & E4 and E2 & E3 are, therefore, parallel in pairs and their weight is ½. The Euler's formula in 2D is E-V=4-4=0, but the normalized formula in 2D is En-Vn=4×½-4×¼=1.

In **3-dimensional space**, the parallelepiped has eight vertices at the following points: (x,y,z)=V1: (0,0,0), V2: (0,0,1), V3: (0,1,0), V4: (0,1,1), V5: (1,0,0), V6: (1,0,1), V7: (1,1,0), V8: (1,1,1).
They are all equivalent by translations and their weight in the normalized Euler's formula is ⅛.
The 12 edges are formed by segments between pairs of vertices that have two coordinates equal, having the following lengths along the x,y,z directions:

E1=V1-V2=(0,0,1)	E2=V1-V3=(0,1,0)	E3=V1-V5=(1,0,0)	E4=V2-V4=(0,1,0)
E5=V2-V6=(1,0,0)	E6=V3-V4=(0,0,1)	E7=V3-V7=(1,0,0)	E8=V4-V8=(1,0,0)
E9=V5-V6=(0,0,1)	E10=V5-V7=(0,1,0)	E11=V6-V8=(0,1,0)	E12=V7-V8=(0,0,1)

The four edges E1, E6, E9, E12 are parallel, as are the quadruples E2, E4, E10, E11 and E3, E5, E7, E8. Each edge has the weight of ¼ in the modified Euler's formula.
The bounding faces of the parallelepiped are formed by corners and edges that have one coordinate in common. Out of $\binom{8}{4}$=1680 combinations only 6 fulfill this condition:

F1=V1/V2/V3/V4 at *x*=0 F2=V5/V6/V7/V8 at *x*=1 F3=V1/V2/V5/V6 at *y*=0
F4=V3/V4/V7/V8 at *y*=1 F5=V1/V3/V5/V7 at *z*=0 F6=V2/V4/V6/V8 at *z*=1

Pairs of faces F1/F2, F3/F4, F5/F6 and F7/F8 are parallel and each face has the weight of ½. Thus in 3D the normalized Euler's formula is Fn-En+Vn=6×½-12×¼+8×⅛=1, whereas the original Euler's formula is F-E+V=6-12+8=2.

In **four dimensions**, the hyper-parallelepiped has 16 equivalent vertices with the following coordinates *w*, *x*, *y*, *z*, each with weight of $^1/_{16}$ in the normalized Euler's formula:

V1: (0,0,0,0) V2: (0,0,0,1) V3: (0,0,1,0) V4: (0,0,1,1),
V5: (0,1,0,0) V6: (0,1,0,1) V7: (0,1,1,0) V8: (0,1,1,1),
V9: (1,0,0,0) V10: (1,0,0,1) V11: (1,0,1,0) V12: (1,0,1,1),
V13: (1,1,0,0) V14: (1,1,0,1) V15: (1,1,1,0) V16: (1,1,1,1)

The bounding edges are those 32 linear segments, i.e. combinations of two vertices that have three coordinates equal. Their lengths are:

E1=V1-V2=(0,0,0,1) E2=V1-V3=(0,0,1,0) E3=V1-V5=(0,1,0,0) E4=V1-V9=(1,0,0,0)
E5=V3-V4=(0,0,0,1) E6=V2-V4=(0,0,1,0) E7=V2-V6=(0,1,0,0) E8=V2-V10=(1,0,0,0)
E9=V3-V7=(0,0,0,1) E10=V3-V11=(0,0,1,0) E11=V4-V8=(0,1,0,0) E12=V4-V12=(1,0,0,0)
E13=V5-V6=(0,0,0,1) E14=V5-V7=(0,0,1,0) E15=V5-V13=(0,1,0,0) E16=V6-V8=(1,0,0,0)
E17=V6-V14=(0,0,0,1) E18=V7-V8=(0,0,1,0) E19=V17-V15=(0,1,0,0) E20=V8-V16=(1,0,0,0)
E21=V9-V10=(0,0,0,1) E22=V9-V11=(0,0,1,0) E23=V9-V13=(0,1,0,0) E24=V10-V12=(1,0,0,0)
E25=V10-V14=(0,0,0,1) E26=V11-V12=(0,0,1,0) E27=V11-V15=(0,1,0,0) E28=V12-V16=(1,0,0,0)
E29=V13-V14=(0,0,0,1) E30=V13-V15=(0,0,1,0) E31=V14-V16=(0,1,0,0) E32=V15-V16=(1,0,0,0)

There are four sets of eight parallel edges that are equivalent by translations, and the weight of each edge in the normalized Euler's formula is ⅛.

The faces, *i.e.* the 2-dimensional bounding elements, each contain four vertices with two common coordinates. There are 24 of them with the following coordinates of their vertices:

F1=V1/V2/V3/V4= (0,0,0,0)/(0,0,0,1)/(0,0,1,0),(0,0,1,1)
F2=V1/V2/V5/V6= (0,0,0,0)/(0,0,0,1)/(0,1,0,0),(0,1,0,1)
F3=V1/V2/V9/V10= (0,0,0,0)/(0,0,0,1)/(1,0,0,0),(1,0,0,1)
F4=V1/V3/V5/V7= (0,0,0,0)/(0,0,1,0)/(0,1,0,0),(0,1,1,0)
F5=V1/V3/V9/V11= (0,0,0,0)/(0,0,1,0)/(1,0,0,0),(1,0,1,0)
F6=V1/V5/V9/V13= (0,0,0,0)/(0,1,0,0)/(1,0,0,0),(1,1,0,0)
F7=V2/V4/V6/V8= (0,0,0,1)/(0,0,1,1)/(0,1,0,1),(0,1,1,1)
F8=V2/V4/V10/V12= (0,0,0,1)/(0,0,1,1)/(1,0,0,1),(1,0,1,1)
F9=V2/V6/V10/V14= (0,0,0,1)/(0,1,0,1)/(1,0,0,1),(1,1,0,1)
F10=V3/V4/V7/V8= (0,0,1,0)/(0,0,1,1)/(0,1,1,0),(0,1,1,1)
F11=V3/V4/V11/V12= (0,0,1,0)/(0,0,1,1)/(1,0,1,0),(1,0,1,1)
F12=V3/V7/V11/V15= (0,0,1,0)/(0,1,1,0)/(1,0,1,0),(1,1,1,0)
F13=V4/V8/V12/V16= (0,0,1,1)/(0,1,1,1)/(1,0,1,1),(1,1,1,1)
F14=V5/V6/V7/V=8 (0,1,0,0)/(0,1,0,1)/(0,1,1,0),(0,1,1,1)
F15=V5/V6/V13/V14= (0,1,0,0)/(0,1,0,1)/(1,1,0,0),(1,1,0,1)
F16=V5/V7/V13/V15= (0,1,0,0)/(0,1,1,0)/(1,1,0,0),(1,1,1,0)
F17=V6/V8/V14/V16= (0,1,0,1)/(0,1,1,1)/(1,1,0,1),(1,1,1,1)
F18=V7/V8/V15/V16= (0,1,1,0)/(0,1,1,1)/(1,1,1,0),(1,1,1,1)
F19=V9/V10/V11/V12= (1,0,0,0)/(1,0,0,1)/(1,0,1,0),(1,0,1,1)
F20=V9/V10/V13/V14= (1,0,0,0)/(1,0,0,1)/(1,1,0,0),(1,1,0,1)
F21=V9/V11/V13/V15= (1,0,0,0)/(1,0,1,0)/(1,1,0,0),(1,1,1,0)
F22=V10/V12/V14/V16= (1,0,0,1)/(1,0,1,1)/(1,1,0,1),(1,1,1,1)
F23=V11/V12/V15/V16= (1,0,1,0)/(1,0,1,1)/(1,1,1,0),(1,1,1,1)
F24=V13/V14/V15/V16= (1,1,0,0)/(1,1,0,1)/(1,1,1,0),(1,1,1,1)

There are six groups, each composed of four faces equivalent by translations: (F1/F14/F19/F24), (F2/F10/F20/F23), (F3/F11/F15/F18), (F4/F7/F21/F22), (F5/F8/F16/F17), (F6/F9/F12/F13), and each face contributes to the modified Euler's formula with a weight of ¼.

The 3-dimensional boundaries of the 4-dimensional hyper-parallelepiped have vertices with one coordinate in common and each is built from six of the faces listed above. There are eight such 3-D bounding parallelepipeds (P) at w=0 or 1, x=0 or 1, y=0 or 1, z=0 or 1, containing the following faces:

P1=(F1/F2/F4/F7/F10/F14)	for	w=0
P2=(F19/F20/F21/F22/F23/F24)	for	w=1
P3=(F1/F3/F5/F8/F11/F19)	for	x=0
P4=(F14/F15/F16/F17/F18/F24)	for	x=1
P5=(F2/F3/F6/F9/F15/F20)	for	y=0
P6=(F10/F11/F12/F13/F18/F23)	for	y=1
P7=(F4/F5/F6/F12/F16/F21)	for	z=0
P8=(F7/F8/F9/F13/F17/F22)	for	z=1

The Euler's formula for this 4-dimensional hyper-parallelepiped is P-F+E-V=8-24+32-16=0, and the normalized Euler's formula is Pn-Fn+En-Vn=$8\times\frac{1}{2}-24\times\frac{1}{4}+32\times\frac{1}{8}-16\times\frac{1}{16}$=4-6+4-1=1.

In summary:

	Result of Euler's summation in	
Number of dimensions	standard formula	normalized formula
1	2	1
2	0	1
3	2	1
4	0	1
